# Current Trends in Pediatric Orthodontics: A Comprehensive Review

**DOI:** 10.7759/cureus.68537

**Published:** 2024-09-03

**Authors:** Abdullah Koaban, Sahar K Al-Harbi, Abdulrahman Z Al-Shehri, Buthainah S Al-Shamri, Maha F Aburazizah, Ghaida H Al-Qahtani, Laila H Al-Wusaybie, Lujain B Alkhalifa, Mesk M Al-Saad, Asaad A Al-Nehab, Ferdoos M Al-Halimi

**Affiliations:** 1 Orthodontics and Dentofacial Orthopaedics, Ministry of Health, Riyadh First Health Cluster, Riyadh, SAU; 2 General Dentistry, College of Dentistry, Dar Al Uloom University, Riyadh, SAU; 3 General Dentistry, King Saud University, Riyadh, SAU; 4 General Dentistry, Alenad Compound Medical, Jouf, SAU; 5 General Dentistry, King Abdulaziz University, Jeddah, SAU; 6 General Dentistry, Farabi Dental College, Jeddah, SAU; 7 Dentistry, Imam Abdulrahman Bin Faisal University, Dammam, SAU

**Keywords:** temporomandibular joint disorders (tmds) in children, clear aligners in mixed dentition, early orthodontic intervention, malocclusion in children, pediatric orthodontics

## Abstract

Pediatric orthodontics is a critical field focusing on the diagnosis, prevention, and treatment of dental and facial irregularities in children. This comprehensive review explores current trends and methodologies in pediatric orthodontics and discusses the multifactorial etiology of malocclusions, including genetic, environmental, and disease-related factors. The importance of proper diagnosis is highlighted, and the extraoral, intraoral, and functional evaluations essential for effective treatment planning are detailed. Various orthodontic conditions such as Class III and Class II malocclusions, abnormal oral habits, arch length discrepancies, anterior and posterior crossbites, open bites, and deep bites are examined in depth. The review also addresses the role of temporomandibular joint disorders (TMDs) and obstructive sleep apnea (OSA) in pediatric patients, emphasizing the need for early and accurate diagnosis to facilitate appropriate intervention. The use of clear aligners in early orthodontic intervention is evaluated given their efficacy and improved patient satisfaction compared to traditional appliances. Additionally, the article discusses the non-advisability of early interception for certain self-correcting malocclusions and the limitations of pediatric orthodontic treatment, including compliance-related issues and the unique anatomical considerations of deciduous dentition. This review aims to provide a detailed understanding of contemporary practices and challenges in pediatric orthodontics, offering insights for clinicians to enhance treatment outcomes and patient care.

## Introduction and background

According to the WHO, malocclusion is one of the top three oral diseases in the world. Malocclusion has a significant impact on growth, development, function, and aesthetics in children. It can impair oral functions, including mastication, speech, swallowing, and temporomandibular joint (TMJ) function. In addition, speech impairment has been reported in children with Class III malocclusion [1,2]. Children with deep bites, open bites, or premature contacts in deciduous or early mixed dentition often show deviations in mandibular movements, subsequently affecting the TMJ [3-5]. Persistent thumb-sucking habits may also alter tongue posture and swallowing patterns in children, contributing to malocclusions such as constricted arches, anterior proclination, and open bite [6,7]. Additionally, malocclusions related to the shape, size, and number of teeth reduce the functional contact area and affect masticatory efficiency [8]. Misaligned teeth will also increase the patient's susceptibility to food debris accumulation and calculus formation, which can lead to caries and periodontal disease [9].

Children with crossbites tend to develop Class III skeletal relationships with a small maxilla and a normal or large mandible. Conversely, a narrow maxilla can restrict mandibular growth and lead to Class II malocclusion [10,11]. Patients with retruded mandibles may develop severe consequences such as sleep apnea, attention deficit hyperactivity disorder (ADHD), and neurocognitive issues [12]. Malocclusion also hampers facial aesthetics, making children vulnerable to bullying [13]. All these factors can significantly impact a child's nutritional intake and therefore overall health and well-being [14].

Pediatric orthodontics, also known as preventive and interceptive orthodontics, is a vital field of dentistry that combines pedodontics and orthodontics. It focuses on fostering dental and oral health at an early age. The primary aim is the early identification and intervention of dental, skeletal, and facial irregularities in children. It involves addressing existing interferences, reducing the need for future orthodontic treatment, and mitigating the severity of developing malocclusion. It also includes educating children and parents about the need for good oral hygiene and regular dental check-ups. According to the American Association of Orthodontists, children are recommended to undergo their first orthodontic evaluation at age 7 or when the first anomaly is noted [15]. Early signs indicating the need for orthodontic intervention include early or late loss of deciduous teeth, increased overjet or overbite, anterior or posterior crossbite, constricted arches, excessive spacing, grinding, or clenching of teeth (bruxism), oral habits (thumb sucking, tongue thrusting, and mouth breathing), improper maxillomandibular relationships, facial asymmetry, speech disorders, difficulty in mastication, facial or dental trauma, supernumerary, ectopic, or missing teeth, and craniofacial anomalies (cleft lip and palate) [16]. The goal of early evaluation is to address problems early, identify and remove the source, guide proper facial development, and provide sufficient space for the eruption of permanent teeth [17].

For clinicians to create individualized and effective treatment plans, it is crucial to understand the etiology behind malocclusion in children. The development of teeth and the maxillofacial complex is influenced by genetic and environmental factors, disease, and trauma [18]. Addressing malocclusion in early mixed dentition proactively may reduce or eliminate the need for future orthodontic treatment. Early orthodontic intervention involves two phases: phase I, performed in early mixed dentition, and phase II, performed in permanent dentition. The two-phase treatment is typically used for skeletal malocclusions, such as Class II and Class III malocclusions.

From a societal and economic perspective, the importance of the early diagnosis of malocclusion in children cannot be overstated. Through early diagnosis, individualized treatment planning, and parental education, pediatric orthodontics plays a pivotal role in ensuring children achieve healthy, beautiful smiles that last a lifetime. However, managing extensive craniofacial deformities and syndromes in children, such as cleft lip and palate, Down's syndrome, hereditary ectodermal dysplasia, as well as malocclusions in mentally challenged patients, requires a multidisciplinary approach involving orthodontists, pedodontists, oral and maxillofacial surgeons, and other medical specialists. Given the intricate nature of these deformities, addressing their diagnosis and treatment plans is beyond the scope of this article. Instead, this review focuses on more general pediatric orthodontic issues, exploring various aspects such as etiology, clinical examination, indications, contraindications, benefits of early orthodontic intervention, various treatment methodologies, latest advancements, and challenges.

## Review

Literature review

A literature search was conducted on indexed journal databases including PubMed, Google Scholar, Scopus, and Cochrane Library. The following search terms for pediatric orthodontics were used: "pediatric orthodontics," "preventive orthodontics," "interceptive orthodontics," "early orthodontic intervention," "occlusal development," "craniofacial development," "oral habits," "cross bite," "class II malocclusion in children," "class III malocclusion in children," "TMDs in children," "OSA in children," "arch length discrepancy," and "dental arch expansion." Boolean operators (i.e., AND, OR, and NOT) were utilized to refine the search. Filters were applied to include only studies in English published between 2005 and 2024. This search strategy ensured the inclusion of current available literature on pediatric orthodontics.

Inclusion criteria

Studies focusing on children between six and 12 years of age, which covers both mixed and early permanent dentition periods, were included. The study designs considered were randomized controlled trials (RCTs), systematic reviews, case-control studies, longitudinal studies, cross-sectional studies, and case reports. Literature from recent edition books was also included. Studies that exclusively discussed interceptive orthodontic treatments (e.g., palatal expansion, myofunctional and orthopedic appliances, habit-breaking, treatment for temporomandibular joint disorders (TMDs) and obstructive sleep apnea (OSA), fixed orthodontics, and aligners) were included. Additionally, studies that reported the outcomes of the treatment (e.g., expansion of arches, correction of malocclusion, elimination of oral habits, reduced need for extraction, improved oral functions such as mastication, speech, and smile, and improved quality of life (QOL) of patients in terms of self-esteem and overall health) were included. Only studies in English published between 2005 and 2024 were considered to take into account recent discoveries. Furthermore, studies published in peer-reviewed journals, studies from diverse geographical locations that provide a global perspective, and studies that obtained ethical committee approval and were available in full text were included.

Exclusion criteria

Studies on children below six years of age, as well as those on adolescent and adult populations, were excluded. Editorials, expert opinions, or reviews without original data were not included. Studies that did not report relevant intervention outcomes, were published before 2005, did not provide clear methodology, were published in languages other than English, were not published in peer-reviewed journals, did not have ethical committee approval, or only had abstracts available were also excluded.

Etiological factors causing malocclusion in children

Malocclusions are typically multifactorial in origin. In children, the development of malocclusion is usually influenced by genetic factors, environmental factors, and diseases [18]. Genetic factors have an impact on the morphology and size of the craniomaxillary complex, whereas environmental factors influence the alveolar and jaw bones [19]. Understanding these etiological factors is essential for effective diagnosis and treatment planning in pediatric orthodontics.

Genetic Factors

Genetic factors are usually associated with race and family history of similar conditions. Certain traits such as jaw, arch, and tooth size are usually inherited genetically [20]. A discrepancy in any of these factors can lead to crowding or spacing. Some malocclusions run in families, such as Class III malocclusions associated with skeletal bases. Therefore, it is highly likely that the child will experience a similar type of malocclusion [21]. Moreover, other inherited craniofacial abnormalities contribute to malocclusions, such as cleft lip and palate. Understanding the importance of genetic influence on malocclusion is crucial since these malocclusions require longer retention and follow-up after early orthodontic treatment [22].

Environmental Factors

Environmental factors include local disorders, diseases, or trauma.

Local disorders: Local disorders influence occlusal development when deciduous teeth are replaced by permanent teeth. The most common environmental factor is deleterious oral habits, such as thumb sucking, tongue thrusting, and mouth breathing. These can lead to the development of anterior open bite, constriction of arches, and proclination of anterior teeth [23]. Another local factor is the early loss or retention of deciduous teeth, which may influence the eruption of permanent teeth [24]. Patients may have the habit of unilateral mastication of food, which can lead to posterior crossbite and ultimately result in facial asymmetry [10].

Diseases:Various diseases may contribute to the development of malocclusion in children. Endocrine disorders, such as hypothyroidism, can affect jaw growth and tooth eruption patterns [25]. Phosphate deficiency can cause rickets and result in delayed tooth eruption, improper alignment of teeth, and abnormalities in jaw development [26]. Patients with Down's syndrome have a higher index of orthodontic treatment need (IOTN) and tend to develop crowding, delayed eruption of teeth, and skeletal Class III malocclusion with poor oral hygiene [27]. Cleidocranial dysplasia leads to multiple impacted teeth along with the development of supernumerary teeth and delayed eruption [28]. Hereditary ectodermal dysplasia affects the number of teeth, as well as their shape and alignment [29]. Patients with cleft lip and palate usually have anterior and posterior crossbites, skeletal Class III malocclusion, missing or impacted teeth, midline deviation, arch length deficiency, crowding, and delayed eruption of teeth [30].

Trauma: Childhood trauma to the dental and maxillofacial region also has a significant impact on the development of normal occlusion and can lead to various types of malocclusions. Trauma can lead to displacement or permanent loss of teeth, ankylosis, root resorption, altered eruption sequence, growth disturbances, and so on [31].

Importance of proper diagnosis in pediatric orthodontics

In pediatric orthodontics, proper diagnosis is crucial to ensure effective treatment planning. To begin, obtaining a detailed history from the parents will provide insights into the child's dental and medical background, familial traits, and behavioral patterns. Parents can offer valuable information about the child's early development, whether the child has habits, such as thumb sucking or mouth breathing, and any previous dental issues or treatments [23]. This information helps orthodontists identify potential genetic predispositions to malocclusion and other orthodontic problems. Additionally, understanding the family's dental history can guide the clinician in predicting growth patterns and planning appropriate interventions [20].

After thorough history-taking, the clinical evaluation of a child is conducted at three levels: extraoral, intraoral, and functional [32]. In the extraoral evaluation, a frontal assessment of the face is done to observe the facial pattern, whether it is dolichocephalic, mesocephalic, or brachycephalic. Another aspect to observe is facial symmetry. If asymmetry is noted, the etiological factor needs to be evaluated, whether it is skeletal or functional. When examining the profile, the child may have a convex, concave, or straight profile, which could be due to abnormal placements of the jaws. The anterior display of the gingiva is also examined to determine whether it is excessive, minimal, or adequate [18].

In the intraoral evaluation, several factors are worth noting. While examining the patient for dental development, they could be ahead or behind for their age. Other anomalies could include aplasia, ectopias, or supernumerary teeth [33]. It is also essential to note the occlusal relationship of molars and canines, whether they are in Class I, II, or III relationships. The bite examination includes whether the patient has a deep bite or open bite and anterior or posterior crossbite (with or without shift). If a posterior crossbite is present, it should be determined if it is unilateral or bilateral. The child should also be observed for maxillary and mandibular midlines to see if they are coincident or not. If they are not, the possible etiological factors should be identified: dental, skeletal, or functional. Another factor to determine is whether the patient has crowding or spacing and, if present, whether it is mild, moderate, or severe. Mild crowding usually resolves on its own, but moderate to severe crowding requires intervention. The frenal attachment also provides valuable information about dental development and speech [34].

Along with clinical evaluation, orthopantomograms (OPG), lateral cephalograms, and frontal cephalograms (if needed to evaluate facial asymmetry) play an important role in accurate diagnosis [35]. This comprehensive approach helps in accurately identifying the underlying causes of malocclusion and planning effective treatment strategies.

Benefits of early orthodontic intervention

Early intervention to treat malocclusion can significantly improve aesthetics and enhance the QOL for patients [36-38]. Addressing malocclusion at an early stage reduces the risk of the condition worsening over time. Furthermore, the skeletal structures in growing children are more adaptable to change, promoting a more rapid orthopedic response. Consequently, early treatment may lessen the duration and complexity of future orthodontic interventions, if needed [39-41].

Moreover, certain conditions, such as anterior and posterior crossbites and abnormal oral habits, are easier to correct when detected early [42-44]. Early detection and management of these issues can prevent more severe complications and contribute to better overall oral health. During the mixed dentition phase, there is typically enough space in the dental arch. If it is identified that the arch length is insufficient to accommodate all permanent teeth, arch expansion can be considered to create additional space. This approach can reduce the need for future extractions of permanent teeth, ensuring a more natural and less invasive treatment process [45,46].

Addressing developing malocclusions in pediatric orthodontics

Class III Malocclusion

Class III malocclusion, also known as mesio-occlusion, is one of the most complicated conditions that an orthodontist can encounter. It is characterized by the mesial relationship of the mandible/mandibular teeth to the maxilla/maxillary teeth. The etiology of Class III malocclusion can be hereditary or environmental, with mandibular growth strongly affected by genes [47]. The hereditary pattern may run in families or occur due to cleft lip and palate or other related craniofacial anomalies [21]. Environmental factors influencing mandibular growth include poor posture (which can alter the position of the condyle in the fossa, causing a forward slide of the mandible), functional shift of the mandible as a result of occlusal interferences or respiratory needs, an abnormally large tongue, cleft lip and palate, an altered airway passage, enlarged adenoids or tonsils, and hormonal imbalances [48,49]. In mixed dentition, this type of skeletal deformity has a prevalence rate of 3.98% [50].

Dental Class III malocclusion typically manifests as an anterior crossbite with a Class I molar relation. Long-standing dental Class III malocclusion, if not addressed early, can interfere with the proper growth of the maxillomandibular complex and result in skeletal Class III malocclusion. The treatment options for dental Class III malocclusion are similar to those for anterior crossbite correction. However, if dental Class III malocclusion occurs due to premature occlusal contacts causing a functional shift of the mandible, it is mandatory to remove interferences as soon as possible.

When treating skeletal Class III malocclusion at an early stage, the main objectives are to improve occlusion, function, and facial aesthetics [51]. Skeletal Class III malocclusion can be classified into three types. Type A includes a normal maxilla and a prognathic mandible (true mandibular prognathism). Type B involves both a prognathic maxilla and mandible, with more prominent mandibular growth. Type C skeletal Class III malocclusion occurs due to a retrognathic maxilla and a normal mandible [52]. Maxillary retrognathism accounts for almost two-thirds of these cases [53]. Several factors must be considered when planning treatment for this type of malocclusion, especially in the mixed dentition phase. The first factor is the patient's growth pattern, whether it is horizontal, vertical, or normal. The second is the amount of anteroposterior discrepancy and the available space for correction. Before initiating intervention, it is crucial to evaluate the patient's compliance since correcting Class III malocclusion at this stage usually requires orthopedic appliances. Therefore, the treatment outcome depends entirely on the patient's cooperation [18].

Growth modification in skeletal Class III malocclusion can be achieved through functional appliances such as FR III or Reverse Twin Block, chin cup therapy, and protraction facemasks with or without expansion. FR III and Twin Block appliances act by enhancing maxillary growth and restricting or possibly redirecting mandibular growth [54]. However, these changes mainly occur due to dentoalveolar changes, not skeletal changes, i.e., proclination of maxillary incisors and retroclination of mandibular incisors. Recent literature shows that FR III corrects Class III malocclusion by restricting mandibular growth, but it does not stimulate maxillary growth [55].

Chin cup therapy can be used in growing children with true prognathic mandibles. It acts by redirecting mandibular growth in a vertical direction, causing clockwise rotation of the mandible [56]. Therefore, it is strictly indicated for patients with skeletal Class III malocclusion and a horizontal growth pattern. However, as true mandibular prognathism tends to reestablish itself once chin cup forces are removed, the therapy is indicated until the patient completes growth [57]. Thus, maxillary protraction with or without palatal expansion is the preferred treatment [58].

A protraction facemask, also known as reverse pull headgear, is usually indicated in skeletal Class III malocclusion with retrognathic maxilla and orthognathic mandible in children below 10 years of age with average or horizontal growth patterns [59]. There is controversy over whether the protraction facemask should be used with maxillary expansion. Expansion is indicated if a retrognathic maxilla is associated with transverse constriction. Initially, expansion with protraction was recommended for all growing skeletal Class III patients as it loosens circummaxillary sutures, promoting the forward growth of the maxilla. However, recent studies have shown equal efficacy of the protraction facemask without expansion [60].

In another study evaluating the effects of early correction of skeletal Class III malocclusion in children between seven and 12 years of age, the authors concluded that there was sufficient evidence to support the positive impact of facemasks on both dental and skeletal components of malocclusion. However, the effects were noted to be of short-term duration [61].

As the protraction facemask is a tooth-borne appliance, it has several unwanted effects on dentition such as extrusion, buccal tilting, and mesialization of maxillary molars. It can also cause an increase in vertical dimensions and loss of arch length. To overcome these unwanted effects, a bone-anchored maxillary protraction (BAMP) appliance has been introduced [62]. In BAMP, miniplates are placed in the infrazygomatic region of the maxilla and the symphyseal region of the mandible. Although BAMP has been found to produce skeletal increments of growth without affecting the maxillary molars, incisors, and growth patterns, it is indicated in patients over 11 years of age. The bone quality needed to provide sufficient anchorage to miniplates is not attained until this age, which may lead to the failure of miniplates. Class III elastics are then extended between these miniplates [63].

Class III malocclusion is a complex and multifactorial condition that requires early intervention and growth modification techniques such as functional appliances, chin cup therapy, and protraction facemask. Understanding the type and severity of the malocclusion is crucial for effective treatment planning.

Class II Malocclusion

Class II malocclusion, also known as disto-occlusion, is the distal relationship of the mandible/mandibular teeth to the maxilla/maxillary teeth. Among all malocclusions, Class II malocclusion accounts for almost 25-30% of cases [64]. The etiology of Class II malocclusion can be either dental or skeletal in origin. Dental Class II malocclusion usually occurs due to the malpositioning of teeth, whereas skeletal Class II malocclusion may present as a prognathic maxilla, a retrognathic mandible, or both. Class II malocclusion can also manifest as a combination of both dental and skeletal factors [65].

Proper diagnosis is crucial when planning treatment for skeletal Class II malocclusion. In cases of Class II malocclusion with a prognathic maxilla, orthopedic appliances such as headgear are recommended. The main aim of using a headgear is to distalize or at least stabilize the maxillary complex [66]. Conversely, if the patient has a normal maxilla with a retrognathic mandible, myofunctional appliances such as activator, bionator, FR II, or Twin Block are indicated depending on the age, dentition stage, teeth alignment, and growth pattern of the patient [67]. Some patients may have both a prognathic maxilla and a retrognathic mandible. In such patients, combination therapy involving both orthopedic and functional appliances can be implemented to restrict the growth of the maxilla and enhance the growth of the mandible [67].

Although early treatment can be initiated for the correction of skeletal Class II malocclusion, there is controversy over whether the treatment should be executed in two phases or only one late phase. Several trials have been conducted to study the effects of growth modification appliances on the mandible [68,69]. However, substantial variations have been observed in treatment outcomes with no reliable predictors. According to clinicians who favor the one-phase treatment, this approach reduces total treatment time, cost, and future consequences such as enamel demineralization or root resorption [70,71]. However, proponents of the two-phase treatment mention several benefits, including a normalized skeletal deformity, improved facial aesthetics, and a shorter phase II [64,72]. Some authors justify initiating early treatment for skeletal Class II to reduce trauma to maxillary incisors, improve the profile, and provide psychological benefits to the patient [73-75].

Most orthopedic and myofunctional appliances require patient compliance. Unless worn regularly, the desired outcome of the treatment is usually hampered. Considering these limitations, the Carriere Motion 3D Appliance (CMA) has received much attention from orthodontists. It is an intermaxillary Class II corrector usually employed in non-extraction cases and consists of a mold-injected rod extending from the maxillary first molar to the maxillary canine. It has a hook on the canine for the engagement of elastics extending from the mandibular molars and a ball-and-socket joint on the maxillary molar to allow for distal tipping and rotation [76]. As Class II malocclusion involves both dental and skeletal factors, treatment options include orthopedic and functional appliances, with the debate ongoing between one-phase and two-phase treatments. Patient compliance is critical for successful outcomes.

Abnormal Oral Habits

Abnormal oral habits play a significant role in the development of malocclusion in children. Common abnormal oral habits are thumb and finger sucking, tongue thrusting, mouth breathing, lip sucking, nail biting, and prolonged use of pacifiers. While these oral habits are normal until 2-4 years of age, it becomes a concern if they persist into the mixed dentition stage as they can then exert abnormal forces on developing dentition and surrounding structures. These abnormal forces will lead to the development of various types of malocclusions. Therefore, these habits should be immediately ceased once detected to prevent deleterious effects and further worsening of malocclusion.

A study was conducted to analyze the relationship between different habits in 106 children aged 5-12 years and various malocclusions in three planes of space. It was found that 72.64% of children had malocclusions in at least one plane of space. Malocclusion in the vertical plane was associated with atypical swallowing and lip sucking; malocclusion in the horizontal plane was a result of oral breathing, atypical swallowing, and digit sucking, while malocclusion in the sagittal plane was associated with the digit-sucking habit. The authors did not find any statistically significant correlation between the sagittal plane and any habit [23].

Another cross-sectional analytical study was conducted among 155 children aged 6-12 years to observe the types of malocclusion in patients with persistent oral habits. The most frequent malocclusion in the vertical plane was a deep bite (22.2%). In the transverse plane, the prevalence of edge-to-edge bite was 7.1% and that of anterior crossbite was 6.5%. In the sagittal plane, 20% of the children had Class II Division 1 malocclusion, and 20.7% had Class III malocclusion. The most deleterious habits were found to be anteroposition (58.7%) and oronasal breathing (51%) [77].

Management of oral habits includes behavioral modification of the child, incorporation of various habit-breaking appliances, and patient and parent education. This helps mitigate permanent adverse effects, promotes proper orofacial development, and prevents the future need for complicated orthodontic treatment. The usual appliances used for the management of tongue thrust are tongue cribs [78].

Orofacial myofunctional therapy (OMT) in the management of oral habits involves the functional training of maxillofacial muscles, especially targeting oral and oropharyngeal muscles. OMT improves muscle tonicity and endurance along with increased coordination between the pharynx and the surrounding area [79]. Clinical studies on OMT have demonstrated beneficial outcomes in swallowing, tongue positioning, and muscle dysfunction [80,81]. Recently, Froggy Mouth, a new myofunctional approach, was tested in 370 patients with atypical swallowing habits. It was found to be effective in correcting oral habits from both clinical and functional perspectives [82]. Another appliance that can be used for the cessation of habits, occlusal guidance, and training of muscle function is the prefabricated myofunctional appliance (Myobrace); it is particularly useful in developing Class II Division 1 malocclusion [83].

Abnormal oral habits such as thumb sucking and tongue thrusting can lead to malocclusions if not intercepted early. Management strategies include behavioral modification, habit-breaking appliances, and myofunctional therapy to promote proper orofacial development.

Arch Length Discrepancies

Arch length discrepancies, also known as Bolton's discrepancy, include insufficient arch length and crowding or excessive arch length and spacing [84]. Several factors contribute to arch length discrepancies. Genetic factors primarily include the size of the jaw bases, craniofacial deformities, congenitally missing teeth, or supernumerary teeth [85]. Environmental factors include early or delayed exfoliation of deciduous teeth, caries, ectopic eruptions, and abnormal oral habits [85]. If arch length discrepancies are effectively managed with early diagnosis and intervention, it can prevent the development of more severe malocclusions in permanent dentition and help establish functional and aesthetically pleasing dentition.

The most common type of arch length discrepancy is crowding [86]. This usually begins with incisor liability, which is 7.6 mm in the upper arch and 6 mm in the lower arch [34]. This may be compensated via interdental spacing between deciduous incisors, the labial eruption of permanent incisors, an increase in the intercanine width, and distal movement of primary canines. As the eruption of lateral incisors is completed, most children show 1-4 mm of crowding in the mandibular anterior region [87]. If incisor crowding is less than 4 mm without any loss of primary teeth, interproximal reduction (IPR) is indicated on the mesiolingual region of primary canines. This provides space for decrowding in the incisor region through tongue pressure, provided there is no root resorption of primary canines due to permanent lateral incisors. This can also be achieved by preserving the E-space, which is done by placing a lower lingual holding arch (LLHA) appliance and either extracting the deciduous first molar or slenderizing the mesial portion of the second molar [88].

Incisor crowding of more than 4 mm usually occurs due to the premature loss of one or more primary teeth. The most common teeth lost prematurely are mandibular deciduous canines [89]. In the maxilla, the space created due to premature exfoliation or loss of teeth is closed by the mesial movement of the teeth distal to the space, whereas in the mandible, closure occurs due to the distal movement of the teeth mesial to the space. Multiple factors can enhance the rate of space closure, including crowding, abnormal oral habits, and deep bites. If there is early exfoliation or loss of primary canines without any space closure, a lingual arch is placed along with a spur distal to lateral incisors to prevent their distalization. However, if the space loss has already occurred but there is space available to regain, a 2×4 appliance is incorporated followed by LLHA for retention. In certain extreme situations, if the space is entirely lost with no scope for space regaining along with a midline shift, the contralateral canine is extracted if present [90]. If both canines are present, serial extraction is indicated [34].

If the primary first molars are lost before the eruption of the permanent first molars, space maintainers are recommended. However, if they are lost after eruption, there is no need for a space maintainer [91]. If the primary second molars are prematurely lost after the eruption of the permanent first molars, a band and loop, a transpalatal arch (TPA), a Nance appliance, or a lower lingual arch is recommended. However, if the second molars are lost before the eruption of the permanent first molars, a distal shoe space maintainer should be placed. The situation becomes complicated if space loss has already occurred after the premature loss of the deciduous second molar and the permanent first molar has erupted in place of the deciduous second molar. This situation demands phase I orthodontic treatment, including space regaining with distalization of the permanent molar through a pendulum appliance, distal jet, or 2×4 appliance with an open coil spring, followed by a Nance button to hold the regained space until the second premolar erupts [88].

Arch length discrepancies, primarily crowding and spacing, are influenced by genetic and environmental factors. Early diagnosis and intervention can prevent severe malocclusions and encourage the development of functional and aesthetically pleasing dentition.

Anterior Crossbites

Anterior crossbites occur when the maxillary incisors are in a lingual relation to the mandibular incisors. If the molars are in a Class I relationship, it is considered to be a dental anterior crossbite. Skeletal anterior crossbites are usually the result of an abnormal basal bone position concerning each other or the cranial base, and they are associated with a Superclass I or Class III molar relationship. Prompt treatment of anterior dental crossbites can prevent attrition of teeth, improve the facial aesthetics of a child, redirect the skeletal growth of the maxilla and mandible, increase the arch perimeter of the maxillary dentition, improve the relationship of teeth to their surrounding alveolar bone, and eliminate potential chances of developing TMDs in the future [92]. If not addressed promptly, it may progress toward a skeletal crossbite.

Anterior crossbites in children are usually treated with removable or fixed appliances. Removable appliances include inclined planes, modified inclined planes, and Hawley's appliances. A normal inclined plane is indicated in patients who have retroclined maxillary incisors, well-aligned mandibular incisors, and average or horizontal growth with or without a functional shift of the mandible [93]. It is cemented on the mandibular anterior teeth considering the degree of overbite. This appliance can correct the crossbite in 3-4 weeks. In patients with retroclined maxillary incisors and proclined mandibular incisors, the inclined plane is modified to cover the incisal edges of mandibular anterior teeth along with Hawley's appliance. This will prevent further proclination of mandibular incisors [94,95]. If there is only palatal tipping of maxillary incisors without bodily movement, a Hawley's appliance with a Z spring or jack screw can be used. It will correct the crossbite in 5-6 weeks [96].

For correcting crossbites in late mixed or early permanent dentition, a 2×4 or 2×6 appliance can be utilized. In this appliance, brackets are bonded to either four or six maxillary anterior teeth, and tubes/bands are placed on maxillary molars. The bite can be raised by placing bite blocks on posterior teeth, which will provide clearance for the correction of the crossbite [97]. Fixed treatment is quicker than removable treatment since patient compliance is not needed.

Anterior crossbites, whether dental or skeletal in origin, should be treated promptly to prevent further complications. Depending on the specific characteristics of the crossbite, treatment options include removable and fixed appliances. An important risk factor for the development of anterior crossbites is tonsillar hypertrophy. Children with tonsillar hypertrophy should therefore be promptly referred to an ENT specialist [97-101].

Posterior Crossbites

Crossbites can be anterior, posterior, or both, with etiologies including dental discrepancy, skeletal discrepancy, or a combination of both. The prevalence of crossbites ranges between 8% and 22% [99]. A posterior crossbite, specifically, is an abnormal buccolingual relationship of premolars and molars in centric occlusion [98,99]. It is recommended to correct posterior crossbites at the earliest opportunity with the help of removable orthodontic appliances. In children with genetic maxillary underdevelopment, protraction headgear or FR III appliances can promote maxillary growth [100].

Posterior crossbites of dental origin are evident due to the tipping or rotation of a tooth or segment of teeth without involving basal bones. Posterior skeletal crossbites involve a combination of lingual inclination of maxillary teeth to mandibular teeth associated with basal bone discrepancy, usually a constricted maxilla with arch length discrepancy [102]. A child may present with either unilateral or bilateral crossbites. Sometimes, a bilateral crossbite creates occlusal interferences in centric occlusion, causing a functional shift of the mandible to one side, clinically presenting as a unilateral crossbite [103]. Posterior crossbites, if not corrected at an early age, may hamper the transverse and anteroposterior growth of the maxillomandibular complex. There is a high chance that this malocclusion will also manifest in permanent dentition, causing facial asymmetries and TMDs [104]. The ideal time to achieve skeletal expansion with long-term stability in insufficient arch width and length or non-coordination of upper and lower arches is from seven to 10 years of age, i.e., before the mid-palatine sutures are fused [105].

Maxillary expansion is the common orthodontic treatment used to correct skeletal posterior crossbites in children. This correction of transverse maxillary deficit can be achieved through either rapid maxillary expansion (RME) or slow maxillary expansion (SME). Both procedures have demonstrated orthopedic effects on maxillary constriction in growing patients [106,107]. RME brings about changes in the maxilla by distributing higher forces in a short period, whereas SME produces lower forces over a longer duration. However, SME has demonstrated more stable and physiologic expansion of maxillary sutures with better bone remodeling [108]. Maxillary expansion occurs at a rate of 0.25-0.5 mm per day until the required expansion is achieved. The typical clinical sign of expansion is the development of a transient midline diastema [97]. Some patients may experience a sensation of pressure at the nasofrontal and other circummaxillary sutures. Slight overexpansion is recommended to accommodate anticipated dental and skeletal relapse. Once the expansion is achieved, bone remineralization in sutures takes place in 3-6 months [109]. If the expander is removed before bone remineralization, it may lead to relapse. Therefore, it must be immediately replaced with retention in some other form, usually a TPA [110].

Various appliances can be used for RME and SME. RME appliances include tooth-borne appliances (Hyrax expander, Isaacson expander) and tooth-tissue-borne appliances (Haas expander, Derichsweiler expander). These appliances can be either bonded or banded. The Inman Power Component (IPC) is another type of RME appliance typically indicated in patients where labial alignment along with maxillary expansion is desired. SME appliances include the quad helix, coffin spring, spring jet, and NiTi expander. However, the placement of banded appliances requires opening up the contacts between teeth with the help of separators, whereas bonded appliances may impinge on the gingiva. To overcome these disadvantages, the MC1 Direct Metal Printed RPE was introduced [111].

Posterior crossbites, whether dental or skeletal, should be corrected early to prevent long-term complications. Treatment options include RME and SME using various appliances, depending on the specific characteristics of the crossbite.

Open Bites

An anterior open bite is characterized by a lack of vertical overlap between the maxillary and mandibular teeth, while the posterior teeth are in contact. It is caused by genetic factors or oral habits and impacts both aesthetics and oral functions. Early intervention with appropriate appliances is essential to prevent further complications.

The etiological factors of an open bite include genetic predisposition or abnormal oral habits, such as prolonged use of pacifiers, thumb sucking, tongue thrusting, or obstruction of the airway due to enlarged adenoids or tonsils [112]. Scientific literature shows that an anterior open bite not only affects the facial aesthetics of a child but also hampers important oral functions such as speech, mastication, and biting and leads to TMDs [113]. Therefore, addressing the open bite as soon as it is detected in a child is critical to prevent severe issues from developing into permanent dentition.

Open bites can be simple, complex, or infantile. The simple/dental/false open bite is limited to the teeth along with surrounding alveolar processes, whereas the complex/skeletal/true open bite is characterized by vertical dysplasia associated with Class I, II, or III malocclusion [114]. However, before addressing the issue, understanding the etiology can help in selecting evidence-based treatment that will effectively manage the condition. In patients with a thumb-sucking habit, palatal cribs or bluegrass appliances are recommended [115,116]. If the open bite has developed due to a tongue-thrusting habit, tongue cribs are used as a physical barrier that prevents the tongue from coming in contact with the anterior teeth. The anterior open bite associated with a constricted maxillary arch is usually accompanied by airway obstruction. In such situations, addressing the reason behind airway obstruction is mandatory before treating the open bite. Once it is managed, the open bite can be treated with functional appliances such as the open bite bionator [117].

Deep Bites

A deep bite is characterized by the excessive overlap of the maxillary anterior teeth over the mandibular anterior teeth. It is a malocclusion where the overbite is more than 4 mm and the maxillary incisors overlap the mandibular incisors by more than 40% [118]. The prevalence of this malocclusion ranges from 5.8% to 18.4% from primary to early mixed dentition [119]. Among all malocclusions, deep bites are less likely to self-correct and have a higher incidence of relapse after orthodontic treatment [120]. If not corrected in childhood, a deep bite can lead to various functional problems such as TMDs, difficulty in mastication, premature wear of dentition, interference with mandibular development, and trauma to the soft tissue of the palate and gingiva [121]. Deep bites may result from either the supra-eruption of maxillary and mandibular incisors or the infra-eruption of posterior teeth [35]. When a deep bite results from the supra-eruption of anterior teeth, it is known as a dentoalveolar/pseudo deep bite, whereas a deep bite resulting from the infra-eruption of posterior teeth is referred to as a skeletal/true deep bite. These can be differentiated using cephalometric analysis.

The etiology of deep bites in children is usually multifactorial, including both genetic and environmental factors. Genetic traits influence underlying dental and skeletal characteristics that contribute to malocclusion. Inherited prognathic maxilla or retrognathic mandible can result in deep bites [122]. Hereditary hyperactivity or hypertrophy of the muscles of mastication and facial muscles can increase the vertical overlap of anterior teeth [123]. Environmental factors include oral habits, diet, early loss of deciduous dentition altering the eruption of permanent dentition, and parafunctional activities such as bruxism [35].

Deep bites can be corrected by extrusion of the posterior teeth, intrusion of the anterior teeth, or a combination of both. However, in the mixed dentition phase, deep bites are usually treated with extrusion of the posterior teeth. One millimeter of extrusion of the posterior teeth causes 2-2.5 mm of bite opening in the anterior region [124]. The appliances of choice are either acrylic bite plane (ABP) or thermoplastic bite plane (TBP). For skeletal deep bites, myofunctional appliances such as activator, bionator, FR, or Twin Block are recommended. These appliances promote the extrusion of posterior teeth and therefore open the bite [122].

Align Technology has recently introduced new treatment options (G8), including specially designed attachments and virtual bite ramps. Attachments, which are composite buttons placed on the buccal surface of teeth, come in various shapes to facilitate tooth movement. They enhance retention and support additional functions, like the use of elastics. Virtual bite ramps operate similarly to bite planes and bite turbos, helping to disocclude the posterior teeth [124,125].

Deep bite malocclusions are characterized by the excessive overlap of anterior teeth and can lead to significant functional problems. Treatment options include extrusion of the posterior teeth and use of various appliances, depending on the specific characteristics of the malocclusion.

TMDs

TMDs are a group of neuromuscular and musculoskeletal disorders involving the TMJ, muscles of mastication, and associated structures. In children, many TMDs go unnoticed because they often have difficulties verbalizing the nature and location of pain and the type of jaw dysfunction [126]. The prevalence rate also varies considerably depending on the number of parents who bring their children to a pedodontist with complaints, the ability of the pedodontist to make an accurate diagnosis, and the diagnostic/examination criteria followed. Gender differences in TMDs are not evident at an early age but become more pronounced between 15 and 50 years, with females being predominant [127]. Therefore, early and accurate diagnosis of TMDs in children plays an important role in treatment planning.

The etiology of TMDs can be divided into predisposing/risk factors, precipitating/initiating factors, and perpetuating/sustaining factors. Predisposing factors include systemic, psychosocial, physiological (cellular, metabolic, neuromuscular), or structural factors (occlusion, craniofacial anomalies). Precipitating factors could be trauma, abnormal posture, or sustained pressure (e.g., from playing wind instruments). Contributing perpetuating factors include parafunctional activities, psychological distress, and occlusal factors [128]. A descriptive cross-sectional study was conducted to examine the association between TMJ sounds and various categories of malocclusions in 384 subjects. Each participant was clinically assessed for TMJ sounds and malocclusion type. No significant association was found between TMJ sounds and different malocclusion categories or gender. TMJ sounds were present in 100 subjects (26%): 22.6% with Class I malocclusion, 32% with Class II, and 31% with Class III. The findings suggested that TMJ sounds are common in healthy individuals without other TMD symptoms and are not indicative of active disease [129].

The typical symptoms of TMDs include orofacial pain, restriction of mouth opening, dental pain, headache, ear pain, and clicking sounds in the joint. The DC/TMD criteria, an international standard for diagnosing TMD, is typically used in adults and can be useful in evaluating TMD symptoms in children as well [130]. TMD can be associated with internal derangement of the disc with or without reduction. These patients may report restricted mouth opening. According to a study, the normal interincisal mouth opening in children is 45 mm [131]. The prevalence of disc displacement associated with restricted mouth opening is 8.3% [132]. In TMD, different mechanical and physiological factors such as repeated stress on retrodiscal tissues, hyperactivity of the muscles of mastication, and abnormal position of the joint can create microtrauma to the tissues, causing ligament elongation and displacement [133].

Managing TMDs in children involves a multidisciplinary approach that focuses on alleviating pain, restoring function, and preventing further damage to the joint. Management also depends on the etiological factors responsible for TMD. Initial treatment typically includes conservative measures such as patient education, behavioral therapy, and physiotherapy to address muscle tension and improve jaw function. Nonsteroidal anti-inflammatory drugs (NSAIDs) may be prescribed to manage pain. In cases where occlusal factors contribute to TMD, orthodontic interventions might be considered. The most common primary orthodontic treatment for pain in TMDs is the occlusal stabilization appliance, which has been proven to be efficient by various studies [134]. It primarily acts by inducing orthopedic stability, minimizing the pathological load on the TMJ, altering the functional relationship, and protecting the surrounding tissues such as the retrodiscal ligament and masticatory muscles [135].

A common condition that can lead to TMDs in children is juvenile idiopathic arthritis (JIA) [136]. Patients with JIA may present with advanced destruction of the TMJ [137], and these patients often develop facial asymmetry, facial deformity, and malocclusion [138]. The treatment involves systemic medication, occlusion stabilization appliances, and intra-articular injections.

TMDs involve the TMJ and associated structures, and they often go unnoticed in children. Early diagnosis and a multidisciplinary approach to management, including conservative measures and orthodontic interventions, are essential for effective treatment.

OSA

OSA involves difficulty breathing during sleep, ranging from snoring to actual obstruction of the airway. It starts as a mouth-breathing habit caused by resistance to nasal breathing. The reason behind nasal breathing could be adenotonsillar hypertrophy (ATH), which alters the tongue position or airway volume [139,140]. Other reasons for altered tongue position can be tongue tie (i.e., an abnormally short lingual frenum) or macroglossia [140,141]. This altered tongue position causes snoring and can lead to OSA [142,143]. Craniofacial deformities responsible for the development of OSA include a constricted maxillary arch with a high or deep palate, maxillary hypoplasia, retrognathic mandible, constricted mandible, vertical growth pattern with steep mandibular and occlusal planes, or a low hyoid bone [143,144]. Other structural abnormalities contributing to OSA are a deviated nasal septum, constricted nasal septum, and hyperplastic nasal conchae [145].

OSA has a significant impact on the systemic health and development of a child. Most of the time, OSA goes undetected in children. It may present as aggressive behaviors or contribute to symptoms of ADHD [146,147]. In children, enlarged adenoids or tonsils are usually treated with adenoidectomy or tonsillectomy [148]. Additionally, children suffering from OSA associated with a constricted maxilla or retrognathic mandible require maxillary expansion and a myofunctional appliance for mandibular advancement [149]. However, it is important to note that not all patients with OSA have craniofacial deformities and not all patients with craniofacial deformities necessarily develop OSA. Therefore, only pediatric patients with OSA and concomitant craniofacial deformity are treatable by orthodontic appliances.

In children with a retruded mandible associated with OSA, mandibular advancement is the best possible option. Mandibular advancement improves OSA by making adaptive changes at the level of the base of the tongue, soft palate, and hyoid bone. This is achieved by modifying the action of genioglossus, palatoglossus, and palatohyoid muscles, which expand the pharynx in all three dimensions, especially the minimal cross-sectional area (MCA) of the pharynx [150]. An increase in MCA enhances oxygen saturation, leading to the restoration of suppressed growth hormone levels, which may resolve restricted mandibular growth [151,152]. The ideal time to start this treatment is 1-2 years before the pubertal growth spurt [153].

OSA with a retruded maxilla is treated with maxillary advancement achieved through a protraction facemask [154], which brings about the downward and forward growth of the maxilla. The nasopharyngeal and velopharyngeal muscles, which are dilators of the upper airway, are attached to the posterior nasal spine (PNS). The downward and forward growth of the maxilla changes the position of the PNS, thereby causing enlargement of these dilator muscles. This leads to forward positioning of the tongue and soft palate, thereby increasing the airway passage [155]. The optimal time to start the protraction of a retrognathic maxilla is between six and nine years of age, before the fusion of circummaxillary sutures [156]. However, if the patient is an adolescent, the protraction of the maxilla can be achieved through a miniplate-assisted facemask or maxillomandibular bone-to-bone traction.

In OSA with nasomaxillary constriction, rapid palatal expansion (RPE) is indicated as a primary treatment option. Along with the forward and downward movement of the maxilla, RPE also brings about the expansion of the entire nasomaxillary complex, including the maxilla, nasal floor, and nasal cavity, in all three dimensions [157]. Expansion of the nasomaxillary complex improves nasal flow, which in turn decreases pharyngeal collapsibility [158]. When combined with a myofunctional appliance in patients with maxillary constriction and a retrognathic mandible, along with nasomaxillary expansion, the treatment also expands the oral cavity by the forward placement of the mandible. This allows forward repositioning of the tongue, thus opening the oropharyngeal airway [159]. Ideally, RPEs should be done before 15 years of age. In young adults, mini-implant-assisted RPE (MARPE) can be used to treat the transverse skeletal discrepancy [160].

Pediatric patients with OSA may have either posterior or total vertical maxillary excess (VME). Posterior VME is characterized by an anterior open bite and tongue thrusting, whereas patients with total VME present with a gummy smile and an anterior deep bite, which locks the mandible in a backward position. VME causes the clockwise rotation of the mandible, decreasing the pharyngeal airway [161]. To correct this craniofacial anomaly, vertical pull headgear is indicated, which restricts the growth of the maxilla. This orthopedic treatment not only increases pharyngeal airway volume but also decreases pharyngeal length [162]. It's indicated in patients below 12 years of age [163].

OSA in children is often related to craniofacial deformities and can significantly impact systemic health and development. Depending on the specific characteristics of the OSA and associated craniofacial deformities, treatment options include maxillary expansion and mandibular advancement.

An overview of pediatric orthodontic conditions is shown in Table 1.

**Table 1:** Overview of pediatric orthodontic conditions TMDs: temporomandibular joint disorders; OSA: obstructive sleep apnea; BAMP: bone-anchored maxillary protraction; RME: rapid maxillary expansion; SME: slow maxillary expansion; NSAIDs: nonsteroidal anti-inflammatory drugs; RPE: rapid palatal expansion

Condition	Etiology	Characteristics	Diagnosis methods	Treatment options
Class III malocclusion	Hereditary, environmental	Mesial relationship of the mandible/teeth to the maxilla/teeth	Clinical examination, cephalometric analysis	Functional appliances (FR III, Reverse Twin Block), chin cup therapy, protraction facemask, BAMP
Class II malocclusion	Dental, skeletal	Distal relationship of the mandible/teeth to the maxilla/teeth	Clinical examination, cephalometric analysis	Headgear, myofunctional appliances (activator, bionator, FR II, Twin Block), Carriere Motion 3D
Abnormal oral habits	Behavioral	Thumb sucking, tongue thrusting, mouth breathing, etc.	Clinical observation, parental interview	Behavioral modification, habit-breaking appliances, myofunctional therapy
Arch length discrepancies	Genetic, environmental	Insufficient or excessive arch length, crowding/spacing	Clinical examination, orthodontic measurements	Early diagnosis, interproximal reduction, space maintainers, lower lingual holding arch appliance
Anterior crossbite	Dental, skeletal	Maxillary incisors lingual to mandibular incisors	Clinical examination, cephalometric analysis	Removable appliances (inclined planes, Hawley's appliance), fixed appliances (2×4 or 2×6 appliance)
Posterior crossbite	Dental, skeletal	Abnormal buccolingual relationship of premolars/molars	Clinical examination, cephalometric analysis	Maxillary expansion (RME, SME), protraction headgear, FR III appliance
Open bite	Genetic, oral habits	Lack of vertical overlap between maxillary and mandibular teeth	Clinical examination, cephalometric analysis	Palatal cribs, bluegrass appliances, tongue cribs, functional appliances (open bite bionator)
Deep bite	Genetic, environmental	Excessive overlap of maxillary anterior teeth over mandibular anterior teeth	Clinical examination, cephalometric analysis	Extrusion of posterior teeth, intrusion of anterior teeth, appliances (acrylic or thermoplastic bite planes)
TMDs	Predisposing, precipitating, and perpetuating factors	Orofacial pain, restriction of mouth opening, dental pain	Clinical examination, DC/TMD criteria, imaging	Conservative measures, NSAIDs, orthodontic interventions, occlusal stabilization appliances
OSA	Craniofacial deformities, oral habits	Difficulty breathing during sleep, snoring	Clinical examination, sleep studies, imaging	Maxillary expansion, mandibular advancement, protraction facemask, RPE

Role of aligners in pediatric orthodontics

Following the above discussion on traditional orthodontic interventions, we will now examine the innovative use of aligners in pediatric orthodontics. This transition from traditional methods to modern techniques demonstrates the evolving nature of orthodontic treatment and the growing preference for less invasive, more comfortable options for children.

Pediatric orthodontics is a controversial topic primarily because of uncertainties regarding its benefits and long-term effects. Early orthodontic intervention in mixed dentition with clear aligners is an interesting yet focal area of pediatric orthodontics. Aligners provide a more advanced approach to managing developing malocclusion in children. They can be considered comfortable alternatives to traditional removable and fixed orthodontic appliances.

A study aimed to evaluate patient and parental satisfaction following mixed dentition treatment with two removable orthodontic devices, i.e., elastodontic appliances (EAs) and clear aligners (CAs), in 56 subjects. The study included an EA group (seven girls and 21 boys; mean age 11 years) and a CA group (12 girls and 16 boys; mean age nine years). A dedicated questionnaire was used to assess the participants' treatment experiences and outcomes. According to both patients and their parents, EAs were significantly more difficult to wear than CAs. Both groups experienced functional improvements, including reduced grinding sounds in the CA group and breathing improvements in the EA group. Parents in the CA group reported significant improvements in school and social life. Additionally, parents in the CA group perceived their child's treatment duration to be much shorter than expected [163]. This study demonstrates the high satisfaction levels associated with the use of clear aligners in pediatric patients.

Clear aligners can also be used to manage arch length discrepancy, which is characterized by the coexistence of primary and permanent dentition with a lack of sufficient space for the proper accommodation of erupting permanent teeth. Aligners function by gradually expanding the dental arch, thus creating the requisite space for proper tooth alignment. A study compared the efficacy and efficiency of clear aligners and a 2×4 appliance for the alignment of maxillary incisor teeth in mixed dentition. The results showed that both methods had similar efficiency and efficacy levels [164]. This confirms that clear aligners are a viable option for treating arch length discrepancies in pediatric orthodontics.

Orthodontic treatment in mixed dentition aims to expand the maxillary arch for proper tooth alignment and to correct sagittal and vertical malocclusions. However, the expected outcomes of these treatments are not well-defined, complicating the standardization of phase I orthodontic treatments. This ambiguity makes it difficult for clinicians to predict tooth movements, including the efficacy of transverse expansion using Invisalign® in children.

The Invisalign First System (First) is a novel orthodontic appliance for maxillary arch expansion in mixed dentition children. Limited studies have compared First with other appliances and included a natural growth group to rule out growth effects. A study evaluated transverse maxillary arch development with the Invisalign First System® in 23 subjects (nine females and 14 males; mean age 9.4±1.2 years). The non-extraction treatment utilized Invisalign First System® clear aligners without additional auxiliaries. Significant increases in maxillary width were observed at the upper first deciduous molars, second deciduous molars, and deciduous canines. The upper first molars exhibited greater expansion in intermolar mesial width compared to intermolar distal and transpalatal width. These results suggest that the Invisalign First System® is effective for maxillary arch development in growing patients, with the most significant expansion observed at the upper first deciduous molars [165].

Another prospective cohort study evaluated the dental and dentoalveolar effects of First versus an acrylic splint RME in adolescents, excluding growth factors. The results showed that both the First and RME groups had significant width increases. The RME group demonstrated greater expansion than the First group in several indicators, including intercanine width and intermolar width. However, there was no significant difference in arch depth between the two treated groups. Both First and RME effectively expanded the maxillary arch in mixed dentition; the RME was more efficient for severe maxillary transverse deficiency (MTD), while First was effective for mild to moderate MTD [166]. These studies indicate that clear aligners, particularly the Invisalign First System, are effective tools for maxillary arch expansion in children.

For the correction of skeletal Class II malocclusion in developing dentition, Invisalign designed a mandibular advancement appliance (MAA) that works on the principle of the Twin Block. It consists of buccally positioned inclined planes, also known as precision wings, in the posterior area of the aligner that positions the mandible forward. MAA has been reported to have multiple advantages over traditional appliances. For example, it can simultaneously align the teeth while correcting the Class II malocclusion. It also has better aesthetics, ease of use, better patient acceptability, and better control over the proclination of incisors and extrusion of molars.

A retrospective cohort clinical study examined the dental, skeletal, and soft tissue effects of MAA in growing patients. All patients had incremental advancements, with the average length of treatment being 9.2 months with an average of 37 aligners. There was a statistically significant difference between the pre-treatment and post-treatment values of the ANB angle, wits appraisal, mandibular length, overjet, overbite, facial convexity, and nasolabial angle [40]. Another study used a cephalometric analysis to compare the dental and skeletal effects of the Van Beek activator, Herbst appliance, Twin Block, and MAA in 63 growing patients with skeletal Class II malocclusion. The results showed that all the appliances successfully achieved mandibular advancements with the correction of Class II molar relationships and deep overjet. However, an increase in lower facial height was unavoidable. Overall, the MAA allowed the aligning and leveling of mandibular anterior teeth with better control over their proclination [167].

In summary, the role of aligners in pediatric orthodontics is expanding, with clear aligners offering a comfortable and effective alternative to traditional appliances. Studies show high satisfaction levels among patients and parents, effective management of arch length discrepancies, and successful maxillary arch expansion and correction of skeletal Class II malocclusions. Aligners, particularly the Invisalign systems, are proving to be valuable tools in early orthodontic intervention and enhance both functional and aesthetic outcomes for pediatric patients. These advancements underscore the potential of aligners to transform pediatric orthodontic treatment by offering significant benefits in terms of patient comfort, treatment efficiency, and clinical outcomes.

A detailed overview of pediatric orthodontic conditions, including their intervention, prognosis, and treatment duration, is shown in Table 2.

**Table 2:** Detailed overview of pediatric orthodontic conditions: intervention, prognosis, and treatment duration TMDs: temporomandibular joint disorders; OSA: obstructive sleep apnea

Condition	Age group for intervention	Prognosis	Complications if untreated	Duration of treatment
Class III malocclusion	Early mixed dentition (6-10 years)	Improved occlusion, function, and aesthetics	Progression to severe skeletal malocclusion	1-2 years
Class II malocclusion	Mixed dentition to early adolescence (7-14 years)	Improved occlusion and facial aesthetics	Increased risk of trauma to maxillary incisors	1-2 years (may vary with treatment phase)
Abnormal oral habits	As soon as detected (2-6 years)	Prevention of malocclusion development	Development of malocclusions	Varies, typically six months to one year
Arch length discrepancies	Mixed dentition (7-11 years)	Functional and aesthetically pleasing dentition	Severe crowding or spacing	1-2 years
Anterior crossbite	Early mixed dentition (6-10 years)	Prevention of attrition, improved aesthetics	Progression to a skeletal crossbite	3-6 months
Posterior crossbite	Early mixed dentition (6-10 years)	Long-term stability, improved occlusion	Transverse and anteroposterior growth issues	Six months to one year
Open bite	Early mixed dentition (6-10 years)	Improved oral functions and aesthetics	Speech issues, TMDs	Six months to one year
Deep bite	Mixed dentition (7-11 years)	Prevention of functional problems	TMDs, trauma to soft tissues	1-2 years
TMDs	As soon as symptoms appear	Pain relief, restored function	Chronic pain, restricted jaw movement	Varies; ongoing management may be needed
OSA	Early childhood (4-10 years)	Improved systemic health, behavior	Systemic health issues, behavioral problems	1-2 years (depending on severity and treatment)
Use of aligners in pediatric orthodontics	Mixed dentition (7-11 years)	High satisfaction, effective management of malocclusions	Potential for incomplete corrections if not monitored	Six months to one year (depending on the condition)

Non-advisability to early interception

When discussing the benefits and advancements of early orthodontic interventions with aligners, it is also essential to understand that not all malocclusions necessitate early treatment. While early intervention can often lead to better outcomes at later stages, there are specific conditions where such procedures are either unnecessary or inadvisable. For instance, a midline diastema measuring less than 2 mm doesn't require any intervention since it is a self-correcting malocclusion that occurs due to the lateral incisor tooth germ pressing against the roots of the central incisors [168]. Distal tipping of lateral incisors is another temporary phase that occurs due to erupting canines, known as Broadbent's phenomenon [168]. Mild crowding in the anterior region usually resolves over time through a labial eruption of permanent incisors, an increase in arch width, and the use of primate spaces [169]. Mild to moderate anterior deep bites are anomalies that can self-correct through the growth of the mandible, increase in alveolar bone height, and molar eruption. Mild distal relationships of the permanent molars get adjusted by the utilization of leeway space.

Limitations to pediatric orthodontic treatment

Certain malocclusions usually do not benefit from early treatment and are prone to relapse at later stages. Early intervention is contraindicated in these malocclusions, such as Class III malocclusions associated with an oversized mandible. Early orthodontic treatment is also not indicated in deciduous dentition even if the patient presents with severe crowding, crossbite, or skeletal discrepancies since children are too young to cooperate. Additionally, deciduous teeth cannot bear orthodontic forces and may exfoliate early.

Children with other medical conditions, such as uncontrolled juvenile diabetes or systemic or autoimmune disorders, are not candidates for early intervention. These patients are prone to repeated infections with delayed healing. Children who cannot maintain proper oral hygiene are at a higher risk of developing caries and periodontal diseases while using orthodontic appliances; therefore, early treatment is contraindicated in such patients.

Uncooperative patients with behavioral issues may not maintain the appliances, causing non-compliance. Interceptive orthodontic treatments are usually deferred until an appropriate time if short-term aesthetics is the primary concern, such as in the case of midline diastema.

Challenges in pediatric orthodontics

Pediatric orthodontics presents a unique set of challenges that necessitate careful decision-making and long-term treatment planning. Children are in a continuous state of growth and development. Some malocclusions in children may not be fully expressed until growth is complete, as these deformities may remain stationary during the active growth period [46]. Additionally, when both genetic and environmental factors are involved in skeletal malocclusion, it can be challenging for clinicians to predict the amount, pattern, and outcome of growth [170]. Dentofacial growth modifications also have certain limitations. The literature provides very limited outcomes of studies done on patients with developing Class III malocclusion due to an oversized mandible [171]. Therefore, extra precautions are advised when deciding on irreversible interventions.

Performing fixed orthodontic treatment on deciduous teeth presents another challenge, as deciduous teeth have short crowns with greater occlusal tapering and an insufficient surface area for bonding and retaining orthodontic appliances. In addition, the roots are short and surrounded by developing alveolar bone that accommodates permanent tooth buds. These delicate structures may not withstand orthodontic forces and may exfoliate early [172]. Persistent oral habits may also impede the expected outcome of the treatment [173]. Moreover, children are not mature enough to adhere to the compliance needed in removable orthodontic therapy and maintaining oral hygiene. This may delay the expected treatment results [174]. As the child continues to grow, long-term follow-up and addressing relapses, if any, are crucial for maintaining the achieved results.

In conclusion, while early orthodontic intervention can be beneficial in many patients, it is not universally advisable. Certain conditions, such as mild midline diastema, distal tipping of lateral incisors, and mild anterior crowding or deep bites, often resolve on their own without intervention. Additionally, early treatment is not recommended for patients with significant behavioral issues, poor oral hygiene, or compromised immune systems. The challenges of pediatric orthodontics, including the unpredictability of growth and the potential for relapse, necessitate careful consideration and individualized treatment planning. These factors underscore the importance of thorough evaluation and cautious decision-making in the management of pediatric malocclusions.

## Conclusions

Pediatric orthodontics is a multifaceted field requiring a comprehensive understanding of the genetic, environmental, and disease-related factors contributing to malocclusions. Accurate diagnosis through detailed extraoral, intraoral, and functional evaluations is crucial for effective treatment planning. Early intervention can significantly improve outcomes for conditions like Class III and Class II malocclusions, abnormal oral habits, arch length discrepancies, crossbites, open bites, and deep bites. TMDs and OSA in children also require timely diagnosis and treatment to prevent long-term complications. Clear aligners are a promising alternative to traditional appliances, offering both comfort and efficacy in early orthodontic intervention. However, not all malocclusions require early treatment, and a thorough evaluation is needed to avoid unnecessary interventions. To improve treatment outcomes, clinicians must address challenges such as patient compliance, anatomical considerations of deciduous teeth, and growth prediction. Continued research and innovation are critical to advancing pediatric orthodontic care and improving dental health and overall well-being among children.
